# Risk factors associated with dengue complications and death: A cohort study in Peru

**DOI:** 10.1371/journal.pone.0305689

**Published:** 2024-06-25

**Authors:** Cesar Copaja-Corzo, Javier Flores-Cohaila, Gustavo Tapia-Sequeiros, Jennifer Vilchez-Cornejo, Miguel Hueda-Zavaleta, Stalin Vilcarromero, Tomas Santana-Téllez, José F. Parodi, Sujey Gomez-Colque, Vicente A. Benites-Zapata

**Affiliations:** 1 Unidad de Investigación para la Generación y Síntesis de Evidencias en Salud, Universidad San Ignacio de Loyola, Lima, Perú; 2 Servicio de Infectología, Hospital Nacional Edgardo Rebagliati Martins, EsSalud, Lima, Perú; 3 Facultad de Ciencias de la Salud, Universidad Científica del Sur, Lima, Perú; 4 Diagnóstico, Tratamiento e Investigación de Enfermedades Infecciosas y Tropicales, Universidad Privada de Tacna, Tacna, Perú; 5 Facultad de Salud Pública y Administración, Unidad de Investigación de Enfermedades Emergentes y Cambio Climático, Universidad Peruana Cayetano Heredia, Lima, Perú; 6 Hospital II Pucallpa, EsSalud, Ucayali, Perú; 7 Facultad de Ciencias de la Salud, Universidad Nacional de Ucayali, Ucayali, Perú; 8 Facultad de Medicina Humana, Centro de Investigación del Envejecimiento (CIEN), Universidad de San Martín de Porres, Lima, Perú; 9 Facultad de Ciencias de la Salud, Universidad Nacional Jorge Basadre Grohmann, Tacna, Perú; Universidad Cooperativa de Colombia, COLOMBIA

## Abstract

**Background:**

Dengue has emerged as an unprecedented epidemic in Peru, and it is anticipated that this issue will escalate further owing to climate change. This study aimed to determine the risk factors associated with death from dengue in patients treated at Hospital II in Pucallpa, Peru.

**Methodology:**

This retrospective cohort study collected information from the medical records of patients with a diagnosis of dengue treated at Hospital II Pucallpa-Peru between January 2019 and March 2023. The primary outcome was death, and the secondary outcome was death, development of severe dengue, or Intensive Care Unit (ICU) admission. Cox regression models were used to determine risk factors.

**Findings:**

The clinical records of 152 patients were evaluated, with a median age of 27.5 years (interquartile range, 11–45). Among all patients, 29 (19.1%) developed severe dengue, 31 (20.4%) were admitted to the ICU, and 13 (8.6%) died during follow-up. In the survival analysis, bilirubin >1.2 mg/dL was associated with a higher risk of death aHR: 11.38 (95% CI: 1.2 106.8). Additionally, factors associated with poor prognosis included having 1 to 3 comorbidities aRR: 1.92 (1.2 to 3.2), AST ≥251 U/L aRR: 6.79 (2.2 to 21.4), history of previous dengue aRR: 1.84 (1.0 to 3.3), and fibrinogen ≥400 mg/dL aRR: 2.23 (1.2 to 4.1).

**Significance:**

Elevated bilirubin was associated with death from dengue, whereas an increase in comorbidities and a history of previous dengue were related to a poor prognosis of the disease. Early identification of severe dengue would be more feasible with improved access to laboratory testing, particularly in tropical areas with a high dengue incidence.

## Introduction

Dengue has left a wave of deaths never seen before in Peru [1]. Dengue is an acute disease transmitted mainly by Aedes Aegypti mosquitoes [2] in tropical and subtropical regions worldwide [3]. The incidence of dengue has dramatically increased worldwide. According to the World Health Organization, an estimated 100–400 million infections occur annually. The disease is endemic in more than 100 countries, including the Americas, which reported 3.1 million cases in 2019, with more than 25 000 classifieds as severe and the largest number of dengue cases ever reported [4].

Peru is a dengue endemic country [5], where more than half of the population is at risk of dengue infection due to 17 of the 25 departments believed to be inhabited by mosquito vectors [6]. In recent years, Peru has faced record numbers of dengue cases, particularly in the Amazon region, which are likely associated with climate change [5]. This is particularly important as Peru experiences an unprecedented heatwave [7], surpassing the historical maximum of 41.1°C in 1963 [8]. This increase in temperature owing to climate change could negatively influence the risk of dengue transmission [9]. This highlights the need for current studies on dengue and other diseases associated with climate change [10].

Although most dengue virus infections are asymptomatic, a wide spectrum of manifestations can occur, ranging from unnoticed infections to severe cases and fatal outcomes [11]. The severe form of the disease is closely associated with significant morbidity and mortality, as reflected in the alarming figure of over 10,000 estimated dengue deaths in 2013 [2,12]. Risk factors such as extreme age, previous dengue infections, and the specific serotype of the virus have been linked to the development of severe cases [13,14].

Two recent systematic reviews have highlighted potential biomarkers associated with an unfavorable prognosis in dengue cases, including elevated levels of C-reactive protein, aspartate aminotransferase, and interleukin 8 and reduced levels of albumin and platelets. However, despite the substantial number of studies included in both reviews, it is important to note that they lacked representation of the Peruvian population, with less than 30% of the included studies conducted in Latin American countries [15,16], demonstrating limited evidence of dengue in Latin America, especially in Peru. Due to the alarming increase in dengue cases in Peru, it is essential to identify markers of poor prognosis for the early detection of patients with poor outcomes. This study aimed to determine the risk factors associated with death and complications of dengue in patients treated at a hospital in Peru.

## Methods

### Study design

We conducted a retrospective cohort study by reviewing the medical records of patients from Hospital II Pucallpa EsSalud from 2019 to 2023. This hospital belongs to the healthcare network of Ucayali and was specifically chosen because of its substantial patient volume, covering 164,318 insured individuals, and its prominence in the Peruvian Amazon as the healthcare facility in Pucallpa, the region’s second most populated city [17]. Our research methodology and manuscript preparation strictly adhered to the Strengthening the Reporting of Observational Studies in Epidemiology (STROBE) guidelines [18].

### Population

For convenience, we performed nonprobabilistic sampling. This method was chosen because we did not have information on all patients who developed severe dengue; therefore, we decided to include all those we identified. Patients with a confirmed diagnosis of dengue were included and identified either by ns1 antigen tests or serological tests (ELISA IgM), depending on the time of illness in which the patient was.

Our focus was on severe cases of dengue, defined by symptoms such as severe plasma leakage causing shock or respiratory distress, significant bleeding, or significant organ dysfunction, including altered consciousness, heart failure, or acute renal failure [11–19]. In addition, we also included patients with dengue with warning signs, defined as persistent vomiting, abdominal pain, clinical fluid accumulation, mucosal bleeding, lethargy or restlessness, liver enlargement <2 cm, and progressive increase in hematocrit with decreased rapid platelet count [11–19]. Patients without laboratory confirmation of dengue, those who were hospitalized during collection, and those referred to another health facility were excluded.

### Data collection and variable definition

We included patients hospitalized between January 1, 2019, and March 1, 2023. We selected this period because, starting in 2019, the medical records at Hospital II Pucallpa EsSalud became digital, allowing for improved patient information retrieval and collection. Patient data were accessed between July 1 and October 30, 2023.

Electronic medical records were retrieved using the Health Intelligence Service, a digital system that archives patient histories, including background information, auxiliary examinations, diagnoses, disease progression, and treatment details. This systematic approach ensured a thorough and consistent data-collection process in our study [20].

Data collection was conducted by two researchers, J.V.C. and G.T.S., who implemented an independent double-data recording system. A third researcher (C.C.C.) reviewed and compared the databases generated by J.V.C. and G.T.S. to ensure equality. When discrepancies were identified, the electronic medical records were reviewed to rectify any errors.

In addition, follow-up of deaths due to dengue was performed during the hospital stay; we considered time zero as admission to the hospital and the final time as the occurrence of death or hospital discharge of the participants.

### Outcome variables

Two outcomes were considered: in-hospital mortality and dengue complications. Hospital mortality was the primary outcome variable. These data were compiled based on the outcomes documented in patients’ medical records throughout the follow-up period.

As a secondary outcome, we assessed dengue complications, which, for the purposes of this study, were defined by the research team as any of the following events: admission to the Intensive Care Unit (ICU), severe dengue or death. These criteria were chosen to comprehensively capture the poor outcomes associated with dengue infections.

### Exposure variables

We categorized the exposure variables into two main groups: clinical characteristics and laboratory parameters. Clinical characteristics included sex (male or female), age (grouped as 7–17, 18–59, and ≥60 years), and comorbidities, which were noted as either present or absent, and further categorized based on the total number of comorbidities (ranging from none to one to three). The laboratory parameters included a range of indicators: leukocytes (1000–3999 / 4000–9999 / ≥10000 cells/mm^3^), lymphocytes (<1500 / ≥1500 cells/mm^3^), neutrophils (<7000 / ≥7000 cells/mm^3^), hemoglobin (<12 / ≥12 g/dL), hematocrit (<45% / ≥45%), platelets (<20,000 / ≥20,000 cells/mm^3^), liver enzymes AST (<50 / 51–250 / ≥251 U/L), ALT (<50 / 51–250 / ≥251 U/L), International Normalized Ratio (INR) (<1.2 / ≥1.2), bilirubin (<1.2 / ≥1.2 mg/dL), creatinine (<1.2 / ≥1.2 mg/dL), urea (<45 mg/dL / ≥45 mg/dL), and fibrinogen (<400 / ≥400 mg/dL). These parameters were selected to comprehensively evaluate the patients’ health status and potential severity of dengue infection [21–24].

### Statistical analysis

We described the variables using frequencies, percentages, and measures of central tendency and dispersion, as appropriate. To identify the factors linked to mortality, we initially applied Cox proportional regression models to each variable. Statistically significant variables (P <0.05) were included in the multivariate model. Similarly, to identify the characteristics associated with disease complications, we utilized Poisson regression with robust variance, incorporating only the multivariate model those variables that were statistically significant (p<0.05) in the raw regression. In both regression models, we decided not to include the urea variable in the multivariate analysis because it had a direct relationship with creatinine (both associated with kidney injury). Therefore, we prioritized the inclusion of the latter. Furthermore, we conducted survival analysis using the Kaplan-Meier method, with the log-rank test deployed to assess differences in survival functions. All analyses were performed using the statistical software Stata v17.

### Ethics

This research project was approved by the Research Ethics Committee of Hospital II Pucallpa EsSalud, as evidenced by the verification code CA-04-CEI-DRAUC-ESSALUD-2023. In adherence to strict confidentiality protocols, all patient data extracted from medical records were anonymized, with random codes assigned to each record for analysis. Given the observational and retrospective nature of our study, informed consent was not sought in line with the ethical guidelines for such research methodologies.

## Results

**Fig 1:**
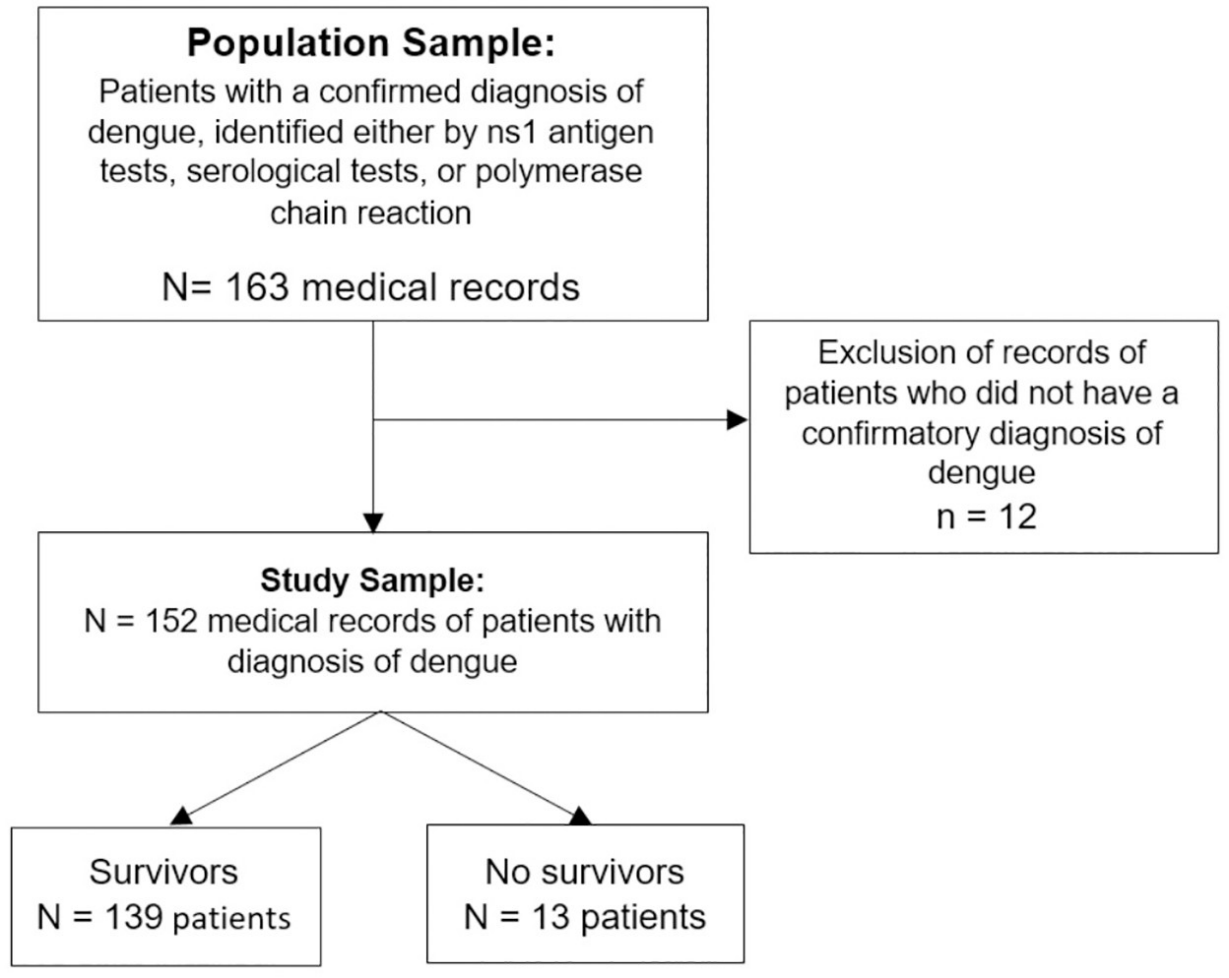
Flowchart detailing the process of sample selection (N = 207).

During the study period, information was collected from 163 medical records. Of these, 12 were excluded because they did not have a confirmed diagnosis of dengue. Finally, 152 medical records of patients with dengue were analyzed (Fig 1).

### Sociodemographic and clinical characteristics

Of the 152 patients, 52.6% were women, and the median age was 27.5 (interquartile range, 11–45) years. Among these patients, 41 (27%) had one–three comorbidities, with high blood pressure (7.5%) and overweight (7.3%). Most patients developed dengue with warning signs (80.9%), and the median length of hospital stay was 6 days (range, 4–9 days).

Regarding clinical characteristics, the most commonly reported symptoms were abdominal pain (75.3%), headache (70.9%), and nausea (70.5%). Most patients (92%) had a Glasgow score of 15 and mean blood pressure of ≥60 mmHg (91.9%). Regarding laboratory characteristics, 74% of the patients had hemoglobin ≥12 g/dL and 87.3% of the patients had a platelet count ≥20,000 cells/mm3 (Table 1).

**Table 1:** Characteristics of the study population (n = 152).

Characteristics	n (%)
Woman sex	80 (52.6)
Age (years)	
7 to 17	63 (41.5)
18 to 59	69 (45.4)
≥ 60	20 (13.2)
Illness duration from one to five days	123 (80.9)
Hospital stay (days)^a^	6 (4 to 9)
Death during follow-up	13 (8.6)
Admission to ICU	31 (20.4)
Development of severe dengue	29 (19.1)
Dengue development with warning signs	123 (80.9)
He went to the emergency room two to three times.	82 (54.0)
History of previous dengue (n* = 135)	39 (28.9)
Dengue IgM Positive (n* = 146)	83 (56.9)
Dengue Ns1 Positive (n* = 145)	92 (63.5)
Comorbidities	
High blood pressure	11 (7.5)
Diabetes Mellitus	10 (7.3)
Overweight	10 (7.3)
Has another comorbidity	21 (13.8)
One to three comorbidities	41 (27.0)
Previous disability	11 (7.2)
Symptoms	
Headache	107 (70.9)
Arthralgia	100 (66.2)
Myalgia	106 (70.2)
Retroocular pain	31 (21.0)
Low back pain	23 (15.7)
Rash	23 (15.7)
Nauseas	105 (70.5)
Threw up	98 (65.3)
Abdominal pain	113 (75.3)
Chest pain	10 (6.9)
Dyspnea	23 (16.0)
Diarrhea	41 (29.7)
Decreased diuresis	15 (10.1)
Mucosal bleeding	35 (23.5)
Signs	
Organ bleeding	13 (8.7)
Mean blood pressure ≥60 mmHg	114 (91.9)
Glasgow coma scale of 15	137 (92.0)
Temperature ≥37.5°C	60 (41.4)
Respiratory rate ≥23 breaths/min	25 (19.7)
Heart rate ≥90 beats/min	58 (43.0)
Laboratory characteristics	
Leukocytes ≥10 000 cells/mm3 (n* = 150)	26 (17.3)
Lymphocytes ≥1500 cells/mm3 (n* = 150)	45 (30.0)
Neutrophils ≥7000 cells/mm3 (n* = 150)	27 (18.0)
Hemoglobin ≥12 g/dL (n* = 150)	111 (74.0)
Hematocrit ≥45% (n* = 150)	29 (19.3)
Platelets ≥20 000 cells/mm3 (n* = 150)	131 (87.3)
AST ≥251 U/L (n* = 123)	32 (26.0)
ALT ≥251 U/L (n* = 108)	17 (15.7)
INR ≥1.2 (n* = 79)	21 (26.6)
Total bilirubin ≥1.2 mg/dL (n* = 105)	20 (20.0)
Creatinine ≥1.2 mg/dL (n* = 89)	16 (18.0)
Urea ≥45 mg/dL (n* = 65)	12 (18.5)
Fibrinogen ≥400 mg/dL (n* = 75)	10 (13.3)

^a^ Median and interquartile range,

n*: Total number of data collected on that variable; ICU: Intensive Care Unit, AST: Aspartate aminotransferase; ALT: Alanine aminotransferase; INR: International Normalized Ratio.

### Factors associated with death from dengue

**Fig 2:**
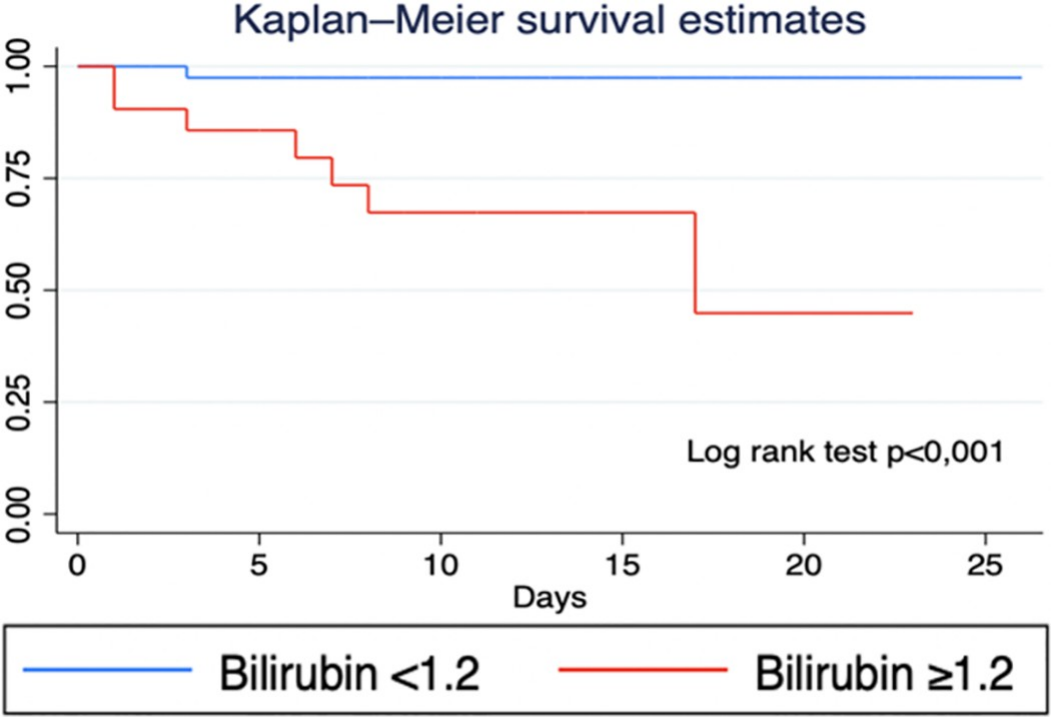
Kaplan-Meier survival curve according to bilirubin category.

In multivariate analysis, bilirubin ≥1.2 mg/dL was associated with a higher risk of mortality (aHR:11.38; IC95%: 1.2 to 106.8) (Table 2). Fig 2 shows the survival function using Kaplan-Meier curves for the variable that was associated with the adjusted regression analysis.

**Table 2:** Characteristics associated with death from dengue.

Characteristics	Crude HR (95% CI)	Adjusted HR (95% CI)
Sex		
Women	Ref	-
Male	0.66 (0.2 to 2.0)	-
Age		
18 to 59 years	Ref	-
7 to 17 years	0.54 (0.1 to 2.2)	-
≥60 years	1.51 (0.4 to 5.6)	-
Comorbidities		
None	Ref	-
One to three	1.48 (0.5 to 4.7)	-
Leukocytes (cells/mm3)		
1000 to 3999	Ref	-
4000 to 9 999	2.04 (6.5 to 6.4)	-
≥10 000	5.26 (NC to NC)	-
Lymphocytes (cells/mm3)		
<1500	Ref	-
≥1500	2.20 (0.7 to 7.3)	-
Neutrophils (cells/mm3)		
<7000	Ref	Ref
≥7000	7.08 (2.1 to 23.9)	5.53 (0.6 to 54.6)
Hemoglobin (g/dL)		
<12	Ref	-
≥12	0.44 (0.1 to 1.4)	-
Hematocrit (%)		
<45	Ref	-
≥45	0.47 (0.1 to 3.7)	-
Platelets (cells/mm3)		
<20 000	Ref	-
≥20 000	1.94 (0.2 to 15.1)	-
AST (U/L)		
<50	Ref	-
51 a 250	1.44 (0.2 to 12.3)	-
≥251	3.46 (0.4 to 30.0)	-
ALT (U/L)		
<50	Ref	-
51 a 250	0.86 (0.2 to 3.3)	-
≥251	0.95 (0.2 to 5.3)	-
INR		
<1.2	Ref	-
≥1.2	3.77 (0.6 to 22.7)	-
Bilirubin (mg/dL)		
<1.2	Ref	Ref
≥1.2	11.31 (2.3 to 55.4)	**11.38 (1.2 to 106.8)**
Creatinine (mg/dL)		
<1.2	Ref	Ref
≥1.2	5.35 (1.4 to 20.2)	0.80 (0.1 to 6.9)
Urea (mg/dL)		
<45	Ref	-
≥45	9.35 (1.7 to 51.2)	-
Fibrinogen (mg/dL)		
<400	Ref	-
≥400	1.81 (0.4 to 8.0)	-

HR, Hazard Ratio, Ref: Reference Variable, AST, aspartate aminotransferase; ALT: Alanine aminotransferase; INR: International Normalized Ratio

### Factors associated with disease complications

In the multivariable model, the characteristics associated with disease complications were having more than one comorbidity (aRR: 1.92; IC95%: 1.2 to 3.2), history of previous dengue (aRR: 1.84; IC95%: 1.0 to 3.3) AST ≥250 U/L (aRR: 6.79; IC95%: 2.2 to 21.4) and fibrinogen level ≥400 mg/dL (aRR: 3.46; IC95%: 1.5 to 8.1) (Table 3).

**Table 3:** Characteristics associated with disease complications.

Characteristics	Uncomplicated dengue (n = 109) n(%)	Complicated dengue (n = 43) n(%)	Crude RR (95% CI)	Adjusted RR (95% CI)
Sex				
Women	60 (75.0)	20 (25.0)	Ref	-
Male	49 (68.1)	23 (31.9)	1.28 (0.8 to 2.1)	-
Age (years)				
18 to 59	43 (68.3)	20 (31.8)	Ref	-
7 to 17	54 (78.3)	15 (21.7)	0.68 (0.4 to 1.2)	-
≥60	12 (60.0)	8 (40.0)	1.26 (0.7 to 2.4)	-
Comorbidities				
None	89 (80.2)	22 (19.8)	Ref	Ref
One to three	20 (48.8)	21 (51.2)	2.58 (1.6 to 4.2)	1.92 (1.2 to 3.2)
History of previous dengue				
No	81 (84.4)	15 (15.6)	Ref	Ref
Yes	23 (59.0)	16 (41.0)	2.63 (1.4 to 4.8)	1.84 (1.0 to 3.3)
Leukocytes (cells/mm3)				
1000 to 3999	41 (87.2)	6 (12.8)	Ref	Ref
4000 to 9 999	58 (75.3)	19 (24.7)	1.93 (0.8 to 4.5)	0.92 (0.3 to 2.6)
≥10 000	9 (34.6)	17 (65.4)	5.12 (2.3 to 11.4)	0.93 (0.2 to 4.7)
Lymphocytes (cells/mm3)				
<1500	80 (76.2)	25 (23.8)	Ref	-
≥1500	28 (62.2)	17 (37.8)	1.59 (1.0 to 2.6)	-
Neutrophils (cells/mm3)				
<7000	99 (80.5)	24 (19.5)	Ref	Ref
≥7000	9 (33.3)	18 (66.7)	3.42 (2.2 to 5.4)	2.22 (0.5 to 10.1)
Hemoglobin (g/dL)				
<12	28 (71.8)	11 (28.2)	Ref	-
≥12	80 (72.1)	31 (27.9)	0.99 (0.6 to 1.8)	-
Hematocrit (%)				
<45	86 (71.1)	35 (28.9)	Ref	-
≥45	22 (75.9)	7 (24.1)	0.83 (0.4 to 1.7)	-
Platelets (cells/mm3)				
<20 000	11 (57.9)	8 (42.1)	Ref	-
≥20 000	97 (74.1)	34 (26.0)	0.62 (0.3 to 1.1)	-
AST (U/L)				
<50	16 (84.2)	3 (15.8)	Ref	Ref
51 a 250	54 (75.0)	18 (25.0)	1.58 (0.5 to 4.8)	2.37 (0.8 to 7.4)
≥251	14 (43.8)	18 (56.3)	**3.56 (1.2 to 10.6)**	**6.79 (2.2 to 21.4)**
ALT (U/L)				
<50	23 (69.7)	10 (30.3)	Ref	-
51 a 250	42 (72.4)	16 (27.6)	0.91 (0.5 to 1.8)	-
≥251	9 (52.9)	8 (47.1)	1.55 (0.8 to 3.2)	-
INR				
<1.2	46 (65.7)	24 (34.3)	Ref	-
≥1.2	3 (33.3)	6 (66.7)	1.60 (0.9 to 2.8)	-
Bilirubin (mg/dL)				
<1.2	39 (67.2)	19 (32.8)	Ref	Ref
≥1.2	10 (47.6)	11 (52.4)	2.55 (1.6 to 4.1)	0.80 (0.4 to 1.6)
Creatinine (mg/dL)				
<1.2	62 (73.8)	22 (26.2)	Ref	Ref
≥1.2	7 (33.3)	14 (66.7)	2.78 (1.9 to 4.1)	1.41 (0.7 to 2.8)
Urea (mg/dL)				
<45	35 (66.0)	18 (34.0)	Ref	-
≥45	2 (16.7)	10 (83.3)	2.45 (1.6 to 3.9)	-
Fibrinogen (mg/dL)				
<400	40 (61.5)	25 (38.5)	Ref	**Ref**
≥400	3 (30.0)	7 (70.0)	1.82 (1.1 to 3.0)	**3.46 (1.5 to 8.1)**

HR, Hazard Ratio, Ref: Reference Variable, AST, aspartate aminotransferase; ALT: Alanine aminotransferase; INR: International Normalized Ratio

## Discussion

To the best of our knowledge, this is the first study in Peru to identify risk factors associated with dengue mortality and complications. A higher number of comorbidities, a history of previous dengue, and liver injury, as indicated by elevated bilirubin and transaminase levels, were the main markers associated with poor disease prognosis.

In fatal cases of dengue, the liver is the most frequently affected organ [25]. Its clinical presentation can be asymptomatic and progress to acute liver failure (ALF) [26]. Although its initial presentation is more commonly associated with elevated transaminase levels, elevated bilirubin is a better marker of ALF [27,28], In our study, it was directly associated with patient mortality, like the findings reported in studies conducted in India [29] and Thailand [30]. In patients with dengue, ALF is a serious complication as it can exacerbate coagulation disorders, including disseminated intravascular coagulation, infection (septicemia), renal failure, increased intracranial pressure leading to cerebral edema, and ultimately cardiopulmonary collapse, resulting in multiple organ failure and patient death [31,32]. Although the pathophysiology of hepatic involvement is not fully understood, it is postulated that hepatic manifestations result from direct viral toxicity or a dysregulated immune response to the virus [25]. This is supported by studies that, when evaluating patients who died from dengue, detected dengue virus RNA and the presence of viral antigens in sinusoidal endothelial cells and Kupffer cells of the liver [25,33]. These findings may explain the hepatic damage observed in patients with dengue.

We found that a higher number of comorbidities was associated with an unfavorable prognosis for dengue. This could be attributed to the fact that the presence of comorbidities complicates the clinical management of patients with dengue, especially in the case of cardiovascular, chronic renal, and pulmonary diseases [34]. Unlike other studies, we did not find that advanced age was associated with a higher death rate from dengue [34–36]. This is probably due to the small number of patients included in our study, which could have reduced the power to detect differences between populations with and without dengue complications.

We observed that elevation of fibrinogen, a key player in the coagulation cascade that contributes to blood clot formation, is associated with a poor prognosis for dengue. The decrease in fibrinogen levels in severe cases of dengue is probably due to dengue infection, which mainly activates fibrinolysis in the absence of a thrombotic stimulus, leading to the direct degradation of fibrinogen and secondary activation of several procoagulant homeostatic mechanisms [37]. Our results contrast with those of other studies that reported that severe forms of dengue with hemorrhagic manifestations present with prolonged clotting times and low fibrinogen levels [37–42]. This discrepancy with our findings could be attributed to the fact that the increase in fibrinogen is secondary to a compensatory response to improve platelet function and reduce the risk of bleeding in patients with severe dengue [43,44]. It is postulated that the development of hemorrhage in patients with dengue may be due to a combination of thrombocytopenia, coagulation imbalances, dysfunctional surviving platelets, and increased fibrinolysis [35,37]. This is probably why we had very few patients with hemorrhagic manifestations of dengue in our cohort.

Climate change will have multiple consequences for health worldwide [45], altering the global distribution of both infectious and zoonotic disease incidences and the risk of these diseases emerging in new regions [46]. "El Niño-Southern Oscillation," the Cyclone "Yacu," and climate change likely contributed to increased vector density [47], due to the accumulation of stagnant water from heavy rains and longer warm seasons [5]; as both conditions favor mosquito proliferation [48], increasing their survival and viral replication [6]. Additionally, it has been associated with bite rates [49], which in turn increases the risk of dengue transmission and other vector-borne diseases [45]. To this end, serious problems of access to clean water and sanitation for rural populations and the poor management of solid waste by regional governments in Peru have been added [50]. It is a combination of these circumstances that has unleashed the largest dengue epidemic in Peruvian history.

Faced with this situation, national governments of Latin America must promote intervention policies supported by scientific evidence to mitigate the probable increase in diseases related to climate change. Our study identified early laboratory markers associated with a poor disease prognosis. Providing access to laboratory tests in areas with a high prevalence of dengue could facilitate the diagnosis and identification of patients with poor prognosis, with the aim of providing them with greater surveillance and timely treatment [51].

This study has certain limitations that must be considered when analyzing the results. The main limitation was its retrospective nature, which prevented the evaluation of certain variables that could be confounding factors or contribute to explaining the study phenomenon (such as self-medication and other concomitant infections). However, owing to the limited resources available, the NS1 and IgM tests for dengue are qualitative in our hospital. This limits the possibility of evaluating whether the quantitative levels of NS1 and IgM are associated with severity. Additionally, owing to the observational nature of our study, we were unable to establish causality between variables. Furthermore, the study was small and was only carried out in one institution; therefore, the results are not generalizable to the entire Peruvian population.

However, this study has important strengths. To the best of our knowledge, this is the first study to identify poor prognostic factors in patients with dengue in Peru. Furthermore, given the evident climate change and possible expansion of dengue in Latin America, especially in Peru, our data are of great relevance. As health services are often limited in resource-poor settings, any information that can help distinguish patients with severe dengue at a higher risk of progressing to death may be crucial.

In conclusion, our study revealed that liver injury, as indicated by an increase in bilirubin level, was the main predictor of mortality in patients with dengue. Additionally, a higher number of comorbidities and a history of dengue were associated with a poor prognosis. Therefore, early identification of dengue patients with poor prognosis could be improved by providing greater access to laboratory testing, particularly in tropical areas with a high prevalence of dengue. This is crucial, considering that dengue has become a significant public health problem worldwide.

## Supporting information

S1 File Study database. (XLSX)

S2 File Supplementary Table 1. (DOCX)

## References

[1] MINSA. Sala situacional de Dengue. https://www.dge.gob.pe/sala-situacional-dengue/#grafico01 (accessed Jan 10, 2024).

[2] Shepard DS, Undurraga EA, Halasa YA, Stanaway JD. The global economic burden of dengue: a systematic analysis. Lancet Infect Dis 2016; 16: 935–41. doi: 10.1016/S1473-3099(16)00146-8 27091092

[3] Simmons CP, Farrar JJ, van Vinh Chau N, Wills B. Dengue. N Engl J Med 2012; 366: 1423–32. doi: 10.1056/NEJMra1110265 22494122

[4] WHO. Dengue and severe dengue. 2023. https://www.who.int/news-room/fact-sheets/detail/dengue-and-severe-dengue (accessed April 16, 2023).

[5] Dengue emergency in the Americas: time for a new continental eradication plan. Lancet Reg Health—Am 2023; 22: 100539. doi: 10.1016/j.lana.2023.100539 37388708 PMC10300565

[6] Dostal T, Meisner J, Munayco C, et al. The effect of weather and climate on dengue outbreak risk in Peru, 2000–2018: A time-series analysis. PLoS Negl Trop Dis 2022; 16: e0010479. doi: 10.1371/journal.pntd.0010479 35771874 PMC9278784

[7] GESTIÓN N. ¿Olas de calor generarían riesgo de mortalidad en Perú?: Esto dice el Senamhi | Fenómeno El Niño | meteorología | | PERU. Gestión. 2023; published online Sept 5. https://gestion.pe/peru/olas-de-calor-generarian-riesgo-de-mortalidad-en-peru-esto-dice-el-senamhi-fenomeno-el-nino-meteorologia-noticia/ (accessed Jan 10, 2024).

[8] Berríos PMR. Arde la selva: Varias ciudades de la Amazonía peruana registrarán cifras históricas de calor sostenido, según Senamhi. infobae. 2023; published online Oct 4. https://www.infobae.com/peru/2023/10/04/arde-la-selva-varias-ciudades-de-la-amazonia-peruana-registraran-cifras-historicas-de-calor-sostenido-segun-senamhi/ (accessed Jan 10, 2024).

[9] Wang Y, Zhao S, Wei Y, et al. Impact of climate change on dengue fever epidemics in South and Southeast Asian settings: A modelling study. Infect Dis Model 2023; 8: 645–55. doi: 10.1016/j.idm.2023.05.008 37440763 PMC10333599

[10] ONU. Los próximos cinco años serán los más cálidos jamás registrados | Noticias ONU. 2023; published online May 17. https://news.un.org/es/story/2023/05/1521047 (accessed Jan 10, 2024).

[11] WHO. Dengue: Guidelines for Diagnosis, Treatment, Prevention and Control: New Edition. Geneva: World Health Organization, 2009 http://www.ncbi.nlm.nih.gov/books/NBK143157/ (accessed April 16, 2023). 23762963

[12] Gutierrez-Barbosa H, Medina-Moreno S, Zapata JC, Chua JV. Dengue Infections in Colombia: Epidemiological Trends of a Hyperendemic Country. Trop Med Infect Dis 2020; 5: 156. doi: 10.3390/tropicalmed5040156 33022908 PMC7709707

[13] Yeh C-Y, Chen P-L, Chuang K-T, et al. Symptoms associated with adverse dengue fever prognoses at the time of reporting in the 2015 dengue outbreak in Taiwan. PLoS Negl Trop Dis 2017; 11: e0006091. doi: 10.1371/journal.pntd.0006091 29211743 PMC5718413

[14] Liew SM, Khoo EM, Ho BK, et al. Dengue in Malaysia: Factors Associated with Dengue Mortality from a National Registry. PloS One 2016; 11: e0157631. doi: 10.1371/journal.pone.0157631 27336440 PMC4919027

[15] Thach TQ, Eisa HG, Hmeda AB, Faraj H, Thuan TM, Abdelrahman MM, et al. Predictive markers for the early prognosis of dengue severity: A systematic review and meta-analysis. PLoS Negl Trop Dis. 2021 Oct 5;15(10):e0009808. doi: 10.1371/journal.pntd.0009808 34610027 PMC8519480

[16] Moallemi S, Lloyd AR, Rodrigo C. Early biomarkers for prediction of severe manifestations of dengue fever: a systematic review and a meta-analysis. Sci Rep 13, 17485 (2023). doi: 10.1038/s41598-023-44559-9 37838744 PMC10576797

[17] EsSalud. EsSalud. 2024. https://portal.essalud.gob.pe/ (accessed Jan 10, 2024).

[18] Vandenbroucke JP, von Elm E, Altman DG, et al. Strengthening the Reporting of Observational Studies in Epidemiology (STROBE): explanation and elaboration. Int J Surg Lond Engl 2014; 12: 1500–24. doi: 10.1016/j.ijsu.2014.07.014 25046751

[19] CDC. Dengue Clinical Presentation | CDC. Cent. Dis. Control Prev. 2023; published online April 13. https://www.cdc.gov/dengue/healthcare-providers/clinical-presentation.html (accessed Jan 10, 2024).

[20] EsSalud W. EsSalud implementa historia clínica digital para atención de asegurados | EsSalud. 2019. http://www.essalud.gob.pe/essalud-implementa-historia-clinica-digital-para-atencion-de-asegurados/ (accessed Jan 10, 2024).

[21] Vincent JL, Moreno R, Takala J, et al. The SOFA (Sepsis-related Organ Failure Assessment) score to describe organ dysfunction/failure. On behalf of the Working Group on Sepsis-Related Problems of the European Society of Intensive Care Medicine. Intensive Care Med 1996; 22: 707–10. doi: 10.1007/BF01709751 8844239

[22] Hasan MJ, Tabassum T, Sharif M, et al. Comparison of clinical manifestation of dengue fever in Bangladesh: an observation over a decade. BMC Infect Dis 2021; 21: 1113. doi: 10.1186/s12879-021-06788-z 34715814 PMC8555248

[23] Jain S, Mittal A, Sharma SK, et al. Predictors of Dengue-Related Mortality and Disease Severity in a Tertiary Care Center in North India. Open Forum Infect Dis 2017; 4: ofx056. doi: 10.1093/ofid/ofx056 28491893 PMC5419201

[24] Mahmood R, Benzadid MdS, Weston S, et al. Dengue outbreak 2019: clinical and laboratory profiles of dengue virus infection in Dhaka city. Heliyon 2021; 7: e07183. doi: 10.1016/j.heliyon.2021.e07183 34141938 PMC8188050

[25] Chagas GCL, Rangel AR, Noronha LM, et al. Risk factors for mortality in patients with dengue: A systematic review and meta-analysis. Trop Med Int Health 2022; 27: 656–68. doi: 10.1111/tmi.13797 35761748

[26] Samanta J, Sharma V. Dengue and its effects on liver. World J Clin Cases WJCC 2015; 3: 125–31. doi: 10.12998/wjcc.v3.i2.125 25685758 PMC4317605

[27] Lambden S, Laterre PF, Levy MM, Francois B. The SOFA score—development, utility and challenges of accurate assessment in clinical trials. Crit Care 2019; 23: 374. doi: 10.1186/s13054-019-2663-7 31775846 PMC6880479

[28] Karoli R, Fatima J, Siddiqi Z, Kazmi KI, Sultania AR. Clinical profile of dengue infection at a teaching hospital in North India. J Infect Dev Ctries 2012; 6: 551–4. doi: 10.3855/jidc.2010 22842941

[29] Prajapati R, Mehta R, Kabrawala M, et al. Dengue hepatitis: Incidence, spectrum and outcome. Indian J Gastroenterol Off J Indian Soc Gastroenterol 2023; 42: 355–60. doi: 10.1007/s12664-023-01405-0 37335522

[30] Teerasarntipan T, Chaiteerakij R, Komolmit P, Tangkijvanich P, Treeprasertsuk S. Acute liver failure and death predictors in patients with dengue-induced severe hepatitis. World J Gastroenterol 2020; 26: 4983–95. doi: 10.3748/wjg.v26.i33.4983 32952344 PMC7476175

[31] Gotthardt D, Riediger C, Weiss KH, et al. Fulminant hepatic failure: etiology and indications for liver transplantation. Nephrol Dial Transplant Off Publ Eur Dial Transpl Assoc—Eur Ren Assoc 2007; 22 Suppl 8: viii5–8. doi: 10.1093/ndt/gfm650 17890263

[32] Gill RQ, Sterling RK. Acute liver failure. J Clin Gastroenterol 2001; 33: 191–8. doi: 10.1097/00004836-200109000-00005 11500606

[33] Marianneau P, Steffan AM, Royer C, et al. Infection of primary cultures of human Kupffer cells by Dengue virus: no viral progeny synthesis, but cytokine production is evident. J Virol 1999; 73: 5201–6. doi: 10.1128/JVI.73.6.5201-5206.1999 10233989 PMC112571

[34] Campos KB, Amâncio FF, de Araújo VEM, Carneiro M. Factors associated with death from dengue in the state of Minas Gerais, Brazil: historical cohort study. Trop Med Int Health 2015; 20: 211–8. doi: 10.1111/tmi.12425 25345964

[35] de Sousa SC, da Silva TAM, Soares AN, Carneiro M, Barbosa DS, Bezerra JMT. Factors associated with deaths from dengue in a city in a metropolitan region in Southeastern Brazil: a case-control study. Rev Soc Bras Med Trop 2022; 55: e0043. doi: 10.1590/0037-8682-0043-2022 36169487 PMC9549950

[36] de Mendonça MFS, de Silva AP SC, Lacerda HR. Factors associated with death from dengue and chikungunya virus infection during an epidemic period in Northeast Brazil: A retrospective cohort study. Rev Soc Bras Med Trop 2023; 56: e0030–2023. doi: 10.1590/0037-8682-0030-2023 37283343 PMC10238066

[37] Wills BA, Oragui EE, Stephens AC, et al. Coagulation Abnormalities in Dengue Hemorrhagic Fever: Serial Investigations in 167 Vietnamese Children with Dengue Shock Syndrome. Clin Infect Dis 2002; 35: 277–85. doi: 10.1086/341410 12115093

[38] Guzman MG, Gubler DJ, Izquierdo A, Martinez E, Halstead SB. Dengue infection. Nat Rev Dis Primer 2016; 2: 1–25. doi: 10.1038/nrdp.2016.55 27534439

[39] Chuang Y-C, Lin Y-S, Liu C-C, et al. Factors contributing to the disturbance of coagulation and fibrinolysis in dengue virus infection. J Formos Med Assoc Taiwan Yi Zhi 2013; 112: 12–7. doi: 10.1016/j.jfma.2012.10.013 23332424

[40] Sureshkumar VK, Vijayan D, Kunhu S, Mohamed Z, Thomas S, Raman M. Thromboelastographic Analysis of Hemostatic Abnormalities in Dengue Patients Admitted in a Multidisciplinary Intensive Care Unit: A Cross-sectional Study. Indian J Crit Care Med Peer-Rev Off Publ Indian Soc Crit Care Med 2018; 22: 238–42. 10.4103/ijccm.IJCCM_486_17 PMC5930527 29743762

[41] Díaz-Quijano FA. [Predictors of spontaneous bleeding in dengue patients: a systematic review of the literature]. Invest Clin 2008; 49: 111–22. 18524337

[42] Whitehorn J, Farrar J. Dengue. Clin Med 2011; 11: 483–7. 10.7861/clinmedicine.11-5-483 PMC4954247 22034713

[43] Brummel-Ziedins KE, Wolberg AS. Global assays of hemostasis. Curr Opin Hematol 2014; 21: 395–403. doi: 10.1097/MOH.0000000000000074 25054908 PMC4163940

[44] Stensballe J, Ostrowski SR, Johansson PI. Viscoelastic guidance of resuscitation. Curr Opin Anesthesiol 2014; 27: 212. doi: 10.1097/ACO.0000000000000051 24514038

[45] Rossati A. Global Warming and Its Health Impact. Int J Occup Environ Med 2017; 8: 7–20. doi: 10.15171/ijoem.2017.963 28051192 PMC6679631

[46] Trejo I, Barnard M, Spencer JA, et al. Changing temperature profiles and the risk of dengue outbreaks. PLOS Clim 2023; 2: e0000115.

[47] CDC-Perú. Boletines epidemiológicos. CDC MINSA. 2024. https://www.dge.gob.pe/portalnuevo/publicaciones/boletines-epidemiologicos/ (accessed Jan 11, 2024).

[48] Terradas G, Manzano-Alvarez J, Vanalli C, Werling K, Cattadori IM, Rasgon JL. Temperature affects viral kinetics and vectorial capacity of Aedes aegypti mosquitoes co-infected with Mayaro and Dengue viruses. BioRxiv Prepr Serv Biol may 2023; 17:2023.05.17.541186. doi: 10.1101/2023.05.17.541186 38374048 PMC10877814

[49] Mordecai EA, Cohen JM, Evans MV, et al. Detecting the impact of temperature on transmission of Zika, dengue, and chikungunya using mechanistic models. PLoS Negl Trop Dis 2017; 11: e0005568. doi: 10.1371/journal.pntd.0005568 28448507 PMC5423694

[50] Copaja-Corzo C, Santana-Téllez TN. Gestión del agua y disminución de reservorios de Aedes Aegypti: Un problema de salud pública sin resolver en Perú. Rev. Cuerpo Med. HNAAA. 11 de junio de 2023;16(1).

[51] Lee RA, Kirby JE. We Cannot Do It Alone: The Intersection of Public Health, Public Policy, and Clinical Microbiology. Clin Lab Med 2019; 39: 499–508. doi: 10.1016/j.cll.2019.05.008 31383271 PMC6686869

