# Supplementary material for: Risk factors associated with dengue complications and death: A cohort study in Peru
Source: PLoS One. 2024 Jun 25;19(6):e0305689. doi: 10.1371/journal.pone.0305689 (PMC11198833; doi:10.1371/journal.pone.0305689)
Supplement: S2 File — (DOCX) [file pone.0305689.s002.docx]

Supplementary table 1: Population characteristics and distribution according to mortality.

| **Characteristics** | **Survivor**  **(n=139)**  **n(%)** | **Non-survivor**  **(n=13)**  **n(%)** |
| --- | --- | --- |
| Sex |  |  |
| Women | 72 (90.0) | 8 (10.0) |
| Male | 67 (93.1) | 5 (6.9) |
| Age |  |  |
| 18 to 59 years | 57 (90.5) | 6 (9.5) |
| 7 to 17 years | 66 (95.7) | 3 (4.4) |
| ≥60 years | 16 (80.0) | 4 (20.0) |
| Comorbidities |  |  |
| None | 104 (93.7) | 7 (6.3) |
| One to three | 35 (85.4) | 6 (14.6) |
| Leukocytes (cells/mm3) |  |  |
| 1000 to 3999 | 47 (100.0) | 0 (0.0) |
| 4000 to 9 999 | 71 (92.2) | 6 (7.8) |
| ≥10 000 | 20 (76.9) | 6 (23.1) |
| Lymphocytes (cells/mm3) |  |  |
| <1500 | 98 (93.3) | 7 (6.7) |
| ≥1500 | 40 (88.9) | 5 (11.1) |
| Neutrophils (cells/mm3) |  |  |
| <7000 | 119 (96.8) | 4 (3.3) |
| ≥7000 | 19 (70.4) | 8 (29.6) |
| Hemoglobin (g/dL) |  |  |
| <12 | 33 (84.6) | 6 (15.4) |
| ≥12 | 105 (94.6) | 6 (5.4) |
| Hematocrit (%) |  |  |
| <45 | 110 (90.9) | 11 (9.1) |
| ≥45 | 28 (96.6) | 1 (3.5) |
| Platelets (cells/mm3) |  |  |
| <20 000 | 18 (94.7) | 1 (5.3) |
| ≥20 000 | 120 (91.6) | 11 (8.4) |
| AST (U/L) |  |  |
| <50 | 18 (94.7) | 1 (5.3) |
| 51 a 250 | 67 (93.1) | 5 (6.9) |
| ≥251 | 27 (84.4) | 5 (15.6) |
| ALT (U/L) |  |  |
| <50 | 29 (87.9) | 4 (12.1) |
| 51 a 250 | 53 (91.4) | 5 (8.6) |
| ≥251 | 15 (88.2) | 2 (11.8) |
| INR |  |  |
| <1.2 | 56 (96.6) | 2 (3.5) |
| ≥1.2 | 18 (85.7) | 3 (14.3) |
| Bilirubin (mg/dL) |  |  |
| <1.2 | 82 (97.6) | 2 (2.4) |
| ≥1.2 | 14 (66.7) | 7 (33.3) |
| Creatinine (mg/dL) |  |  |
| <1.2 | 69 (94.5) | 4 (5.5) |
| ≥1.2 | 11 (68.8) | 5 (31.3) |
| Urea (mg/dL) |  |  |
| <45 | 51 (96.2) | 2 (3.8) |
| ≥45 | 8 (66.7) | 4 (33.3) |
| Fibrinogen (mg/dL) |  |  |
| <400 | 59 (90.8) | 6 (9.2) |
| ≥400 | 7 (70.0) | 3 (30.0) |

AST: aspartate aminotransferase, ALT: Alanine aminotransferase, INR: International Normalized Ratio
